# The Efficacy of Elonide Nasal Corticosteroids in Managing Allergic Rhinitis: A Randomized, Double-Blinded Trial

**DOI:** 10.3390/jcm13071883

**Published:** 2024-03-25

**Authors:** Hardip S. Gendeh, Aneeza W. Hamizan, Salina Husain, Azmawati M. Nawi, Farah D. Zahedi, Nur Fadhilah Megat Ismail, N. Ammal M. Farit

**Affiliations:** 1 Department of Otorhinolaryngology, Head and Neck Surgery, Faculty of Medicine, Universiti Kebangsaan Malaysia, Kuala Lumpur 56000, Malaysia; draneeza@gmail.com (A.W.H.); drsalina_h@yahoo.com (S.H.); farahdayana@ukm.edu.my (F.D.Z.); fadhilahmegat@gmail.com (N.F.M.I.); 2 Allergic Unit, Hospital Canselor Tuanku Muhriz, Jalan Yaacob Latif, Bandar Tun Razak, Cheras, Kuala Lumpur 56000, Malaysia; azmawati@ppukm.ukm.edu.my; 3 Department of Public Health Medicine, Faculty of Medicine, Universiti Kebangsaan Malaysia, Kuala Lumpur 56000, Malaysia; 4 Department of Pharmacy, Faculty of Medicine, Universiti Kebangsaan Malaysia, Kuala Lumpur 56000, Malaysia; ammalfarit@ppukm.ukm.edu.my

**Keywords:** mometasone furoate, nasal spray, Nasonex, non-inferior, side effects

## Abstract

**Background**: Mometasone furoate nasal spray is efficacious in relieving allergic rhinitis symptoms. The objectives of this study were, firstly, to compare the efficacy of Elonide to Nasonex^®^ and a placebo and secondly, to investigate the side effects of Elonide. **Method**: This was a prospective, single-centered, double blinded, randomized, placebo-controlled, non-inferiority trial. A total of 163 participants from the Otorhinolaryngology Clinic, Hospital Canselor Tuanku Muhriz (HCTM), were randomized into three treatment groups receiving Elonide (n = 56), Nasonex^®^ (n = 54), and placebo (n = 53) nasal sprays using an online randomizer (Random.org). Treatment was administered for 4 weeks. The primary outcome measure was the Total Nasal Resistance (TNR), and the secondary outcomes were the Visual Analogue Score (VAS) and the Rhinoconjunctivitis Quality of Life Questionnaire (RQOLQ) score. Side effects were recorded. **Results**: There were significant improvements for all groups from baseline. The Elonide group had the greatest mean difference for all primary and secondary outcomes compared to Nasonex^®^ and the placebo (0.77 ± 2.44 vs. 0.35 ± 1.16, *p* = 1.00 vs. 0.17 ± 0.82, *p* = 0.01). Elonide is non-inferior to Nasonex (*p* = 1.00) and superior to the placebo (*p* < 0.05). The highest side effects reported were for Nasonex (n = 14, 26%), followed by the placebo (n = 8, 16%) and Elonide (n = 6, 12%); headaches (n = 9, 17%) and sore throat (n = 9, 17%) were the most common. **Conclusions**: Elonide has similar efficacy to Nasonex^®^ when compared to a placebo in the treatment of AR in adults. Elonide is safe and tolerable, with fewer side effects and no adverse side effects.

## 1. Introduction

Allergic rhinitis (AR) is an Immunoglobulin (Ig) E-mediated inflammation of the nose upon exposure to previously sensitized allergens [1]. It has a prevalence of almost 40% in the population [2]. Rhinitis symptoms include nasal congestion, runny nose (rhinorrhea), nasal itchiness, and sneezing [3,4]. Severity can be classified into mild and moderate/severe, while its frequency is classified as intermittent and persistent [1]. Allergan avoidance is the key form of management but is impractical as some allergens such as house dust mites and food simply cannot be eradicated or avoided. Long-term pharmacotherapy is required to control symptoms. Newer intranasal corticosteroids such as mometasone furoate (Nasonex^®^) and fluticasone furoate are effective in managing nasal symptoms as they inhibit the T cell response and interleukins 4 and 5 [5,6]. These second-generation nasal steroids are safe, with low levels of systemic absorption.

Mometasone furoate nasal spray (MFNS), first manufactured as Nasonex^®^ (Merck Sharp & Dohme Corp., Heist-op-den-Berg, Belgium), was granted approval in 1997 for the treatment of allergic rhinitis in children (2–11 years) and adults (12 years and above). Several alternatives to Nasonex^®^ are now available in a generic form since its patent ended in December 2017. Elonide (HOE Pharmaceuticals Sdn. Bhd, Petaling Jaya Malaysia) is a generic nasal spray containing mometasone furoate. Elonide is a more affordable substitute to Nasonex^®^ for the treatment of allergic rhinitis. There have been previous studies comparing generic mometasone furoate to Nasonex^®^, and the tested generic medication has a similar therapeutic profile and safety compared to Nasonex^®^ [7,8]. A study comparing 72 patients on generic mometasone furoate to 72 patients with its original and 36 receiving a placebo revealed mean changes in the Total Nasal Symptoms Score (TNSS) of −4.30, −4.59, and −1.93 at two weeks, indicating therapeutic equivalence between the generic and original [9].

To our knowledge, there has been no head-to-head study that compares Elonide and Nasonex^®^ in the treatment of allergic rhinitis. Thus, the objective of this study was to compare the efficacy of the MFNSs Elonide and Nasonex^®^ versus a placebo among AR patients. The secondary objective was to assess the side effects of Elonide compared to Nasonex^®^.

## 2. Materials and Methods

This study was granted approval by the Research Ethics Committee of UKM (UKM PPI/111/8/JEP-2021-655) and ClinicalTrials.gov Identifier (NCT05912192). All participants provided a written informed consent. This study has fulfilled the CONSORT Checklist. Trial registration ClinicalTrials.gov Identifier: NCT05912192 Funded by Hoe Pharmaceuticals Sdn Bhd, Selangor, Malaysia.

### 2.1. Study Design

This was a prospective, single-centered, double-blinded, randomized, placebo-controlled trial conducted at the Department of Otorhinolaryngology, Head and Neck Surgery, Faculty of Medicine, Hospital Canselor Tuanku Muhriz, Universiti Kebangsaan Malaysia.

### 2.2. Participants

Eligible participants included adults aged 18 and above with newly diagnosed mild, moderate-to-severe, intermittent, and persistent allergic rhinitis, as defined by AR and its Impact on Asthma (ARIA) [10]. AR was confirmed either via a positive skin prick test or serum IgE toward at least one allergen [4]. A specific IgE serum level of ≥0.35 kU/L was deemed a positive result [11]. Participants were excluded if they had concomitant asthma, other immunodeficiency diseases, pregnancy, another concomitant rhinology disease, were smokers, had a severely deviated nasal septum, any form of malignancy or ciliary dyskinesia such as cystic fibrosis. Use of the following medications were not permitted 30 days before the initial dosing: antihistamines, products that contain nasal corticosteroids and decongestants.

### 2.3. Intervention

There were three arms involving generic MFNSs (Elonide, 50 mcg/dose, Hoe Pharmaceutical Sdn. Bhd., Petaling Jaya, Malaysia): the original mometasone furoate aqueous nasal spray (Nasonex^®^, 50 mcg/dose, Merck Sharp, & Dohme Corp. Heist-op-den-Berg, Belgium) and a placebo nasal spray (sodium chloride 0.9%, 50 mcg/dose). The pharmacist packaged nasal sprays using aluminum foil to disguise the bottle labels, performed randomization, and dispensed all three nasal sprays. The pharmacist was responsible for generating a random allocation sequence and labeling the bottles sequentially from number 1 to number 163. Investigators and participants were blinded to the type of spray given. Participants were randomized to one of the three groups of intervention. Randomization was performed using an online randomizer (Random.org).

**Figure 1:**
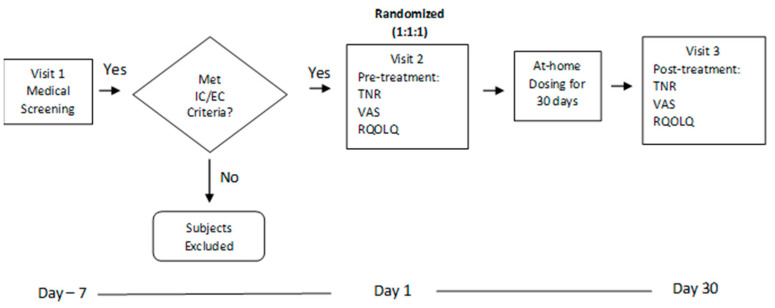
Study flow chart. During the at-home period, for 30 days, patients self-administered the drug studied with doses approximately 12 h apart. The patients’ diaries were used to record symptom assessments and compliancy. IC, inclusion criteria; EC, exclusion criteria; TNR, Total Nasal Resistance; VAS, Visual Analogue Score; RQOLQ, Rhinoconjunctivitis Quality of Life Questionnaire.

This study consisted of a pretreatment visit, 30 days of at-home dosing, and a post-treatment visit on day 30 (Figure 1). During the pretreatment visit, patients were screened for eligibility by the presentation of nasal symptoms for AR as per ARIA. Consent was obtained before patients were randomized into one of the treatment arms (Elonide, Nasonex^®^, or the placebo). They were informed of common side effects of MFNSs and non-efficacious treatment. Primary and secondary outcome tests were performed during the pretreatment and post-treatment visits. Participants were provided one bottle of nasal spray and advised to self-administer one spray per nostril twice daily. Drug administration and symptoms were recorded in a medication diary given. During the 30 days of home administration, compliance was ensured via a follow-up interview and a review of patient diaries. Patients were permitted to have a rescue medication (a loratadine tablet, 10 mg, at maximum once daily) if their symptoms were not tolerable.

### 2.4. Outcome Measures

The efficacy of the nasal corticosteroids was evaluated using the Visual Analogue Score (VAS, mm), the Rhinoconjunctivitis Quality of Life Questionnaire (RQOLQ), and the Total Nasal Resistance (TNR, Pa/cm^3^/s). The assessments were carried out during pretreatment and post-treatment visits. The severity of nasal symptoms via VAS ranged from 0 (least severe) to 100 mm (most severe). The RQOLQ with 7 subdomains (sleep symptoms, non-hay symptoms, practical problems, nasal problems, eye problems, and emotional problems) was used to assess quality of life. The nasal response was objectively assessed by measuring the TNR using an NR6 Rhinomanometer medical instrument (GM Instruments Ltd., Irvine, UK). The primary outcome was the TNR measurement value, and the secondary outcomes were the VAS and the RQOLQ score.

### 2.5. Sample Size

The sample size was calculated using Power and Sample Size Software 3.1 (Dupont & Plummer, 1990). The study looked into a continuous response variable from an independent control and experimental subjects with one control(s) per experimental subject. Based on Sriram et al., 2005, the response within each subject group was normally distributed with a standard deviation (SD) of 0.21 [8]. Suppose the true difference in the experimental and control means is 0.14. Therefore, 36 experimental subjects and 36 control subjects are needed to reject the null hypothesis that the population means of the experimental and control groups are equal with a probability (power) of 0.8. The Type I error probability associated with the test of this null hypothesis is 0.05. Considering a 20% dropout rate, a total of 43 patients were required for each treatment group.

### 2.6. Statistical Analysis

Efficacy analyses of this study were based on an intention-to-treat population, defined as all randomized participants who received one dose or more of the intervention and completed one efficacy assessment or more during treatment [12]. Safety assessments were based on the safety population, which included all participants who received one dose or more of the study intervention. Baseline demographic variables and adverse effects were summarized by treatment group using descriptive statistics. Changes from the baseline to the mean post treatment for all outcomes were examined using an analysis of variance (ANOVA) with the baseline as a covariate and the treatment as fixed effect. The baseline was defined as the mean symptom score during visit 2 prior to 1 month without any intervention, immediately before the first dose (day 1). A treatment difference of *p* < 0.05 was considered statistically significant. The onset of action was defined as the first time point after initiation of treatment, when the intervention demonstrated a significant difference in primary outcomes compared with the placebo as long as the significant difference was sustained. the onset was analyzed using an ANOVA, with the baseline as a covariate and the treatment as a fixed effect. All tests were 2-sided, using a significance level of 5%, and all analyses were conducted using SPSS version 22 and higher.

## 3. Results

### 3.1. Participants

**Figure 2:**
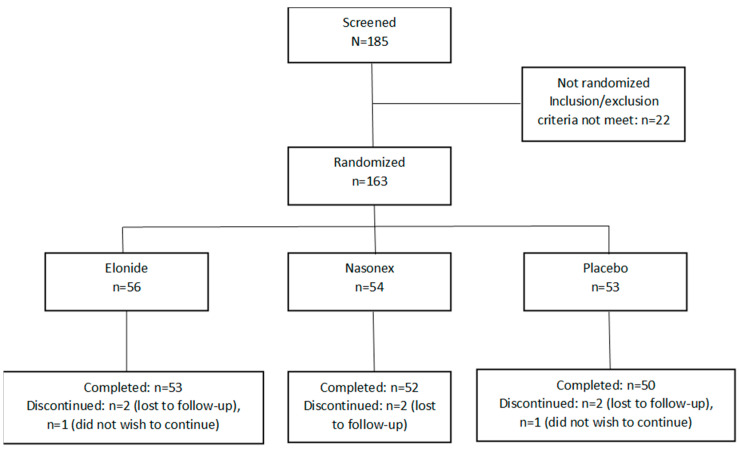
Flow of patients in the study design.

There were 163 participants who were randomized into three treatment groups: Elonide (n = 56), Nasonex^®^ (n = 54), and a placebo nasal spray (n = 53). Eight participants (4.9%) dropped out, leaving 155 participants who completed the study with satisfactory diary documentation (Figure 2). The reasons for dropout were defaulting on follow-up (n = 6) and a reluctance to continue (n = 2) (Table 1). There were 51 male (31%) and 112 female (69%) subjects, with a mean age of 31.23 (SD 8.29) years (Table 1). There were 157 patients that used a nasal spray solely without antihistamines, Elonide (n = 55), Nasonex^®^ (n = 54), and a placebo (n = 48). Rescue medication use was 0.61% (n = 1) in the Elonide group, 0% (n = 0) in the Nasonex^®^ group, and 3.07% (n = 5) in the placebo group. There was no difference in baseline characteristics between groups (Table 1).

### 3.2. Efficacy Outcomes

**Figure 3:**
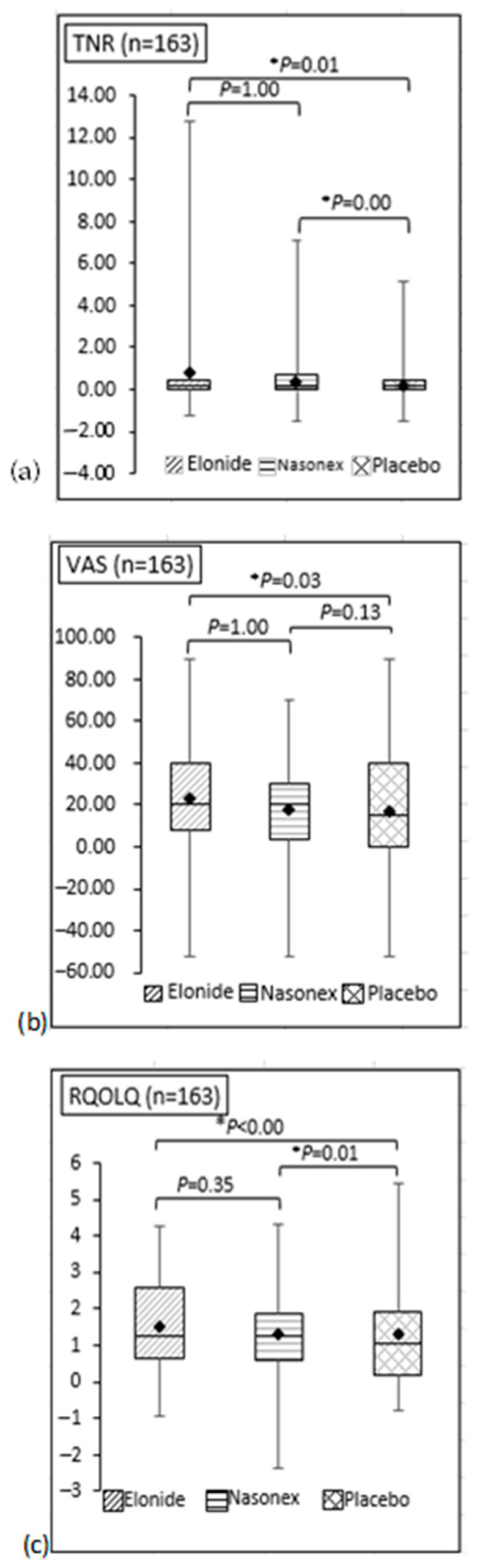
Data distribution with mean plots (♦) for (**a**) TNR (**b**) VAS, and (**c**) RQOLQ score with *p* values. * indicates significant *p* value (*p* < 0.05).

There were improvements in the TNR, VAS, and RQOLQ scores at post treatment within all three groups (Table 2). The mean differences for Elonide were higher than for Nasonex and the placebo for the TNR, the VAS, and the RQOLQ score (Figure 3). Elonide showed a larger improvement in mean values post treatment compared to pretreatment than Nasonex^®^ and placebo. There was a significant difference between Elonide and the placebo (*p* < 0.05). Elonide showed the same efficacy as Nasonex (*p* > 0.05) and is superior to the placebo (*p* < 0.05) (Figure 3).

A post hoc subgroup analyses of the primary end point showed that there was no statistically significant difference between the treatment effect in participants with imputation variables. The imputation variables do not affect the mean difference’s significance. The results were favorable for the actively treated groups (Elonide and Nasonex^®^) compared to the placebo for all the end points which were statistically significant.

### 3.3. Safety Outcomes

All treatments showed similar safety profiles with low incidences of side effects (SEs) (Table 3). Side effects related to treatment were headaches, sore throat, cough, nasal dryness, nasal irritation, epistaxis, and imbalance; side effects were reported as mild in most cases. The percentage of participants reporting one or more SEs was larger in the Nasonex^®^ group. The overall SE incidence was 54%, with headaches and sore throat being common. There were no adverse effects.

## 4. Discussion

Previous studies have found that INC sprays are effective in relieving AR symptoms [13,14,15,16]. Based on ARIA guidelines from 2021, INCs are indicated as first-line treatment for mild (VAS < 5) to severe (VAS > 5) intermittent or persistent AR [10,17,18,19]. The guidelines also suggest a step-up treatment if patients are still symptomatic [10,20,21]. The VAS, RQOLQ score, and TNR showed that Elonide had the same efficacy compared to Nasonex^®^ via both primary and secondary outcomes. This is consistent with previous studies in which a generic MFNS was compared to Nasonex^®^ with similar therapeutic profiles (2 weeks of improvements in the Total Nasal Symptoms and mini-RQLQ scores) and safety, with fewer SEs while relieving AR symptoms [7,8]. This proved that the generic Elonide was constantly as efficacious in treating AR as Nasonex.

Post-treatment assessments were performed at week 4; this is an optimal duration for AR treatment, taking into account that the maximum effect of INCs requires 2 weeks or more [22,23]. Durations beyond 4 weeks have been shown to be non-efficacious for drug testing in perineal allergic rhinitis [22]. The VAS is an appropriate tool for severity evaluations of AR to guide treatment [24,25,26,27,28]. According to ARIA, changes in the VAS greater than 23 mm can be considered of clinical importance and thus reflect responsiveness to treatment [26]. In this study, Elonide had the highest mean difference from baseline (>23 mm), indicating an efficacious response to treatment. However, it was also observed that the mean differences in the VAS from baseline scores were relatively low for this study (Elonide, 23.41 mm; Nasonex^®^, 17.36 mm; and placebo, 16.96 mm) versus a 29 mm mean difference reported by Demoly et al., 2013 [26]. This could be due to patients’ exposure to allergens (dust mites and food) during the long 4-week period causing a temporary worsening of symptoms prior to the post-treatment visit.

The RQOLQ is a valid, reliable, and reproducible disease-specific global tool on the impact of AR used to evaluate QOL [29,30,31]. Higher scores recorded on the RQOLQ are associated with greater QOL impairment. The minimal-importance clinical difference for the RQOLQ score was 0.5 [29]. The mean difference from baseline for the RQOLQ scores further reduced after 4 weeks (>0.5) for Elonide, Nasonex^®^, and the placebo to 1.50, 1.32, and 1.29 respectively, with the highest significant mean difference recorded for Elonide. This is consistent with previous studies that have shown improvements in QOL at 2 weeks and 4 weeks of treatment using MFNSs [16,32]. QOL improvements are key as they affect the severity of AR and compliancy. Furthermore, patients must not only depend on INCs for symptomatic improvements; allergen avoidance (where possible) is a mainstay of treatment for AR that should be practiced to ensure better QOL improvements [33].

Rhinomanometry is an objective test used to access the severity of nasal congestion by measuring the difference in the trans-nasal pressure of air flow through the nasal cavity. Its physiological function is to determine the nasal air flow resistance via a pressure gradient over four consecutive breaths. The normal value of nasal resistance in an adult is 0.25 Pa/cm^3^. This number was taken from previous studies in order to establish a quantitative measure whereby nasal resistance is indicative of nasal obstruction [34,35,36,37]. A lower the flow rate and a higher nasal resistance value indicate a higher degree of nasal obstruction. Therefore, an improvement in rhinomanometry translates into a reduction in nasal congestion [37]. Patients with severe nasal congestion will have a higher TNR, which agrees with current study in which the mean baseline TNR scores for each treatment group were 1.37, 1.17, and 0.81 Pa/cm^3^/s. The Elonide group showed a higher mean difference from baseline, which indicates a reduction in nasal congestion.

INCs of varying brands have different flow properties known as thixotrophy rates [38]. Nasonex^®^ is an aqueous suspension of a corticosteroid which becomes less viscous when shaken or sprayed and returns to a more viscous state within the nasal passages. The Nasonex^®^ group experienced the highest rate of SEs (26%) compared to Elonide (12%) and the placebo (16%). A good aqueous suspension is one that can cover a larger surface area upon spraying, after which it adheres to the mucosa, resulting in less retrograde flow to the nose and throat discomfort. Elonide should have similar properties to Nasonex^®^ as they share the same content of mometasone furoate [39]. However, Elonide not an aqueous solution and should have a higher viscosity, requiring it to be shaken prior to use. A more viscous solution may adhere to the mucosa and be less likely to flow to the back of the nose. This may be a reason for the fewer SEs of sore throat and cough among the Elonide group. There have yet to be any studies comparing flow properties between both of these INCs.

Mometasone furoate has been shown to be efficacious in reducing nasal symptoms such as nasal blockage, nasal itchiness, sneezing, and rhinorrhea for both seasonal and perineal allergic rhinitis [15,40]. It also has the highest lipophilicity compared to fluticasone propionate, beclomethasone dipropionate, and budesonide, resulting in higher uptake within the nasal mucosa [41]. The side effects of MFNSs from previous studies ranged from 22% to 36% [5,14,42,43] wherein headache is the most common, consistent with the findings of this study. Both the generic and original MFNSs were well tolerated with no adverse drug reactions, further confirming their safety profiles. [14,18,42,43]. This is consistent with previous studies on Nasonex^®^ which showed tolerable adverse events or side effects within 2 to 4 weeks of the study’s duration. There were no adverse drug reactions up to 6 months of outpatient follow-up of the above patients outside the study period [44,45,46].

The placebo had significant improvements for the VAS, the RQLQ score, and the TNR when compared to baseline due to the therapeutic effect of normal saline in relieving AR symptoms [47]. Meta-analyses showed that saline irrigation served as a safe adjunct to AR treatment among children and adults [48,49]. A meta-analysis looked into patient-reported disease severity, wherein normal saline was shown to have a therapeutic effect in relieving symptoms for up to 4 weeks (SMD −1.32, 95% confidence interval (CI) −1.84 to −0.81; 407 participants) in six studies and three months (SMD −1.44, 95% CI −2.39 to −0.48; 167 participants) in five studies [50]. Although normal saline 0.9% was used as a placebo, previous AR-related research studies did not state the type of placebo used [7,8,13,14,15,16]. Five patients within the placebo group required oral antihistamines as rescue medications daily and/or when needed. Since allergen avoidance is a mainstay of treatment, exposure to allergens may also affect the outcomes measured between groups. Therefore, future studies should consider the use of distilled water instead of normal saline as a placebo as the latter has therapeutic benefits.

Multiple imputation (MI) was conducted for missing data at random to preserve the randomized sample size (n = 163) [51]. MI is a popular method of imputing data in randomized clinical trials via predictive mean matching (PMM). It analyses data by imputing missing data for continuous variables and is less sensitive to violations of the normality assumption compared to standard linear regression imputation [52,53]. More than five imputations are performed to avoid a large Monte Carlo error [52]. The number of imputations should be at least greater than the percentage of missing data analyses; the rate of missing data for this study was 4.9%, equivalent to five imputations used [54].

The limitation of this study was a small sample size due to challenges with recruiting newly diagnosed patients without prior INC administration. A cross-over study with a washout period should be considered in the future. Classifying the severity of AR may also allow treatment outcomes among different groups to be analyzed. A short follow-up period is a limitation. Therefore, a longer duration of follow-up within the study period beyond 4 weeks can be considered for better monitoring of adverse events or side effects with time. A lower value of *p* < 0.05 was not used to indicate significance as results should be interpreted in combination and not depending solely on the *p* value. Elonide showed greater improvements in mean values for the TNR, the RQLQ score, and the VAS compared to Nasonex^®^ and the placebo [55,56]. The generic version of the MFNS may be a viable alternative to its original counterpart which may be more affordable for developing nations void of national healthcare financing and with patients making high out-of-pocket payments [57].

## 5. Conclusions

Elonide nasal spray has a similar efficacy and is non-inferior when compared to its original counterpart, Nasonex^®^, in the treatment of AR in adults. Elonide is safe, tolerable, has fewer side effects with no adverse reactions; it is suitable as a single-modality treatment to improve nasal symptoms.

## Figures and Tables

**Table 1:** Demographic and baseline characteristics (safety population).

Characteristics	Total	Elonide	Nasonex	Placebo	*p* Value
Subjects randomized to treatment, No. (%)	163 (100)	56 (34)	54 (33)	53 (33)	-
Age, mean (SD), (y)	31.23 (8.29)	32.16 (8.61)	31.57 (8.41)	29.91 (7.78)	0.25
Gender, No. (%)					
Male	51 (31.29)	19 (33.93)	16 (29.63)	16 (30.19)	0.87
Female	112 (68.71)	37 (66.07)	38 (70.37)	37 (69.81)	
Intent to treat (ITT)	163	56	54	53	-
Subjects completed treatment	155	53	52	50	-
Subjects discontinued treatment, No. (%)	8 (4.9)	3 (1.84)	2 (1.23)	3 (1.84)	-
Reason for discontinuation:					
Lost to follow-up	6	2	2	2	-
Did not wish to continue	2	1	0	1	-
Medication(s):					
Nasal spray only, No. (%)	157 (96.32)	55 (33.74)	54 (33.13)	48 (29.45)	-
Nasal spray + antihistamine, No. (%)	6 (3.68)	1 (0.61)	0 (0)	5 (3.07)	-
VAS (mm), mean (SD) ᵃ	56.32 (19.74)	57.09 (17.14)	54.61 (20.71)	57.25 (21.38)	0.71
RQOLQ, mean (SD) ᵃ	2.98 (1.30)	2.87 (1.17)	2.91 (1.38)	3.15 (1.36)	0.49
TNR (Pa/cm^3^/s), mean (SD) ᵃ	1.12 (1.62)	1.37 (2.55)	1.17 (1.48)	0.81 (0.83)	0.26

Abbreviations: VAS, Visual Analogue Score; RQOLQ, Rhinoconjunctivitis Quality of Life Questionnaire; TNR, Total Nasal Resistance. Nasal sprays were dosed as one puff twice daily; ᵃ intent-to-treat population.

**Table 2:** Mean (SD) values for pretreatment and post-treatment (intent-to-treat population) within respective groups.

Treatment Group	Outcome Measures	Pretreatment	Post-Treatment	*p* Value
Elonide	TNR (Pa/cm^3^/s)	1.37 (2.55)	0.56 (0.35)	<0.01
	VAS (mm)	57.09 (17.14)	33.90 (17.80)	<0.01
	RQOLQ	2.87 (1.17)	1.39 (0.95)	<0.01
Nasonex^®^	TNR (Pa/cm^3^/s)	1.17 (1.48)	0.77 (0.60)	<0.01
	VAS (mm)	54.61 (20.71)	37.04 (21.73)	<0.01
	RQOLQ	2.91 (1.38)	1.59 (1.22)	<0.01
Placebo	TNR (Pa/cm^3^/s)	0.81 (0.83)	0.64 (0.40)	<0.01
	VAS (mm)	57.25 (21.38)	40.51 (21.48)	<0.01
	RQOLQ	3.15 (1.36)	1.88 (1.39)	<0.01

Abbreviations: VAS, Visual Analogue Score; RQOLQ, Rhinoconjunctivitis Quality of Life Questionnaire; TNR, Total Nasal Resistance. Nasal sprays were dosed as one puff twice daily.

**Table 3:** Number of subjects (%) with side effects considered to be related to treatment.

Treatment Group				
Side Effects	Elonide (n = 56)	Nasonex^®^ (n = 54)	Placebo (n = 53)	Total
Headaches	2 (4%)	4 (7%)	3 (6%)	9 (17%)
Sore throat	2 (4%)	4 (7%)	3 (6%)	9 (17%)
Cough	1 (2%)	2 (4%)	1 (2%)	4 (8%)
Nasal dryness	1 (2%)	1 (2%)	0 (0%)	2 (4%)
Nasal irritation	0 (0%)	1 (2%)	1 (2%)	2 (4%)
Epistaxis	0 (0%)	1 (2%)	0 (0%)	1 (2%)
Imbalance	0 (0%)	1 (2%)	0 (0%)	1 (2%)
Total	6 (12%)	14 (26%)	8 (16%)	28 (54%)

## Data Availability

All additional data and materials are available on request.
